# COVID-19 vaccines and immunization in the paediatric population

**DOI:** 10.3126/nje.v12i1.43158

**Published:** 2022-03-31

**Authors:** Indrajit Banerjee, Jared Robinson, Brijesh Sathian

**Affiliations:** 1, 2 Sir Seewoosagur Ramgoolam Medical College, Belle Rive, Mauritius; 3 Geriatrics and long term care Department, Rumailah Hospital, Hamad Medical Corporation, Doha, Qatar

## Background

The worldwide SARS-CoV-2 pandemic has forever altered the manner in which the world and various health agencies will deal with such epidemics and pandemics in future. The synthesis of the COVID-19 vaccinations and the various immunization drives which are underway, mainly garnish and confer protection to the adult populous; namely those aged above 18 years in age [1]. The development and further acceptance of vaccinations in the younger paediatric age group is comparatively sedate [2]. UNICEF data released thus far states that 0.4% of the deaths experienced due to COVID-19 (12300) have been recorded in those aged 20 years and below, with 58% of the deaths occurring in those aged 10-19 years and 42% in those aged 0-9 years old respectively [3]. The frustrating and crippling element still causing global COVID- related difficulties is that of the catastrophic mutations and or discoveries which pose a risk to the ongoing global vaccine stratagem. The discovery of these hybrid mutations and how the international community may respond and circumnavigate such events will determine the course for the following round of immunizations [4,5]. The evidence supporting the use of immunization in the general populous stands true, however the use thereof in the paediatric population is not clear and thus the vital question which remains to be definitively answered and explored is that of vaccination in the younger paediatric age groups, namely those aged between 5 and 12 years. The deaths registered in this young populous are high and it is the duty of physicians to protect those whom are weakest through the use of the most scientifically accepted and proven methods [6,7].

The Centre for disease control (CDC) recommends 2 doses of Pfizer vaccine separated by 21 days in those aged 5 to 11 years, with an additional third booster in immunocompromised children [8].

### Current vaccines on the market

The WHO (World Health Organization) has published a list of all the recognized as well as vaccines undergoing the WHO EUL/PQ (emergency use/pre-qualification) evaluation process. What is vitally important to understand is that all vaccines are not the same and do not confer the same level of immunity and protection. The majority of the vaccines are developed for those 18 years of age and above. The various vaccines within the WHO EUL/PQ list are in different levels of testing and are under different phases of study. Among the list of 13 vaccines whom have applied for the WHO approval only 17 thus far have attained the final level of acceptance. These vaccines namely being:

- BNT162b2/COMIRNATY Tozinameran (INN)[EMA,USFDA]
- AZD1222 Vaxzevria [EMA, MFDS KOREA, Japan MHLW/PMDA, Australia TGA, COFEPRIS (Mexico) ANMAT (Argentina)]
- Covishield (ChAdOx1_nCoV-19) [EMA], mRNA-1273[EMA, USFDA, MFDS]
- COVID-19 Vaccine (Vero Cell)
- Inactivated/ Coronavac
- SARS-CoV-2 Vaccine Inactivated (Vero Cell)/ COVAXIN
- NVX-CoV2373/Covovax
- NVX-CoV2373/Nuvaxovid

The earliest acceptance being Pfizer Biotech with its first site approved on the 31st of December 2020. Pfizer’s vaccine was quickly followed by the likes of AstraZeneca, Covishield, Janssen and Moderna Biotech. Bharat Biotech India received the approval for its Whole-Virion Inactivated Vero Cell vaccine on the 3 of November 2021, in contrast to the initial mRNA based vaccines which received earlier approval. It is widely accepted that the mRNA based vaccines have a greater efficacy and therefore have been more widely used by developed nations in their national rollout of vaccinations. The importance of the above list is the fact that only 2 of the above vaccines (Pfizer and Moderna) have received the necessary licenses and have undergone the necessary studies to be approved for the administration in the younger age group. If one looks at the timeline above, it is evident that both Pfizer and Moderna are both mRNA vaccines and were among the first two companies to bring the vaccine to market. This first to market advantage has allowed for the collection of further data as well as funding for greater research to best produce enhanced vaccines for both the adult and now younger paediatric age group and it is thus only sensible that these two pharmaceutical giants are at the forefront of developing these immunizations which are being targeted towards the younger paediatric age group [9-12].

#### Age of vaccination- WHO guidelines

As with all new drugs and vaccines, strict trials and studies must be undergone before the medicament can be applied and acceptably used on the general populous. The same regulations apply for the COVID-19 vaccinations. The initial release of the first vaccines were strictly for groups aged 18 years and above. It is now stated by the WHO that all of the vaccines which have received the WHO EUL are safe for the majority of individuals aged 18 years and above including individuals with pre-existing conditions. The younger age groups were also not naturally targeted in the first round of vaccinations due to the illness causing milder symptoms in the young. It has however now been concluded by the World Health Organizations SAGE (Strategic Advisory Group of Experts) that the Moderna vaccine can be safely used in children aged 12 years and above. The WHO’S SAGE has also concluded that the Pfizer vaccine can be administered safely to children aged 5 years and above.[8] The Centre for disease control (CDC) recommends 2 doses of the Pfizer vaccine separated by 21 days in those aged 5 to 11 years, with an additional third booster in immunocompromised children [13].

### COVID-19 paediatric immunization Nepal

The developing nation of Nepal has received both consignments of COVAX as well Moderna vaccines. The national immunization stratagem aims to vaccinate 1.74 million children aged 12 to 17 years with the Moderna vaccine and used the COVAX vaccine for those aged 18 years and above. This planning and forethought will ensure a rapid rise in herd immunity again the virus via vaccinating from the proverbial “top” and “bottom” of the demographic populous, thus conferring coverage to both the elderly and the young [14,15].

### Vaccination for those aged 6 months to 5 years

Pfizer is currently undergoing a phase 1, dose-finding study in combination with an ongoing phase 2–3 randomized trial which are being used to study the safety and efficacy of a two-dose regimen of the BNT162b2 vaccine. It is a trial on children between 6 months to 11 years old with a 2-dose regimen of 10, 20 and 30 microgram injections. The doses being given at an interval of 3 weeks. The study concluded that a vaccine regimen of two 10 microgram doses of BNT162b2 administered 21 days apart was found to be safe and immunogenic in children 5 to 11 years of age. Pfizer is also under taking a study using immune-bridging techniques in the younger age group between 6 months to 5 years. The study testing a 2-dose regimen of 3 microgram injections; equating to a dose one tenth of that used in adults. The doses being given at an interval of 3 weeks. Early reports suggest that the immune-conversion and response in the younger patients is insufficient and thus may call for a 3-dose regimen. More data is needed in order to categorically determine the efficacy of the vaccines in those under 5 years of age. It is however evident that vaccinations in the younger paediatric age group namely 6 months to 5 years will materialize and it is now a question of when and not if; the premise of vaccinations being accepted in this age group relies on the data which will be produced via the trials currently underway [16-18].

## Conclusion

The efficacy of the COVID-19 vaccines is evident and their role is vital in combatting the mortality, morbidity and development of new mutations. The role of approved vaccines such as Pfizer and Moderna in the younger age groups namely 12 to 18 years of age and 5 to 12 years of age is vital and shows a categorical increase in immunity and protection. It is thus advised for the 5 to 18 year old cohort to receive their COVID-19 vaccinations. The efficacy of such vaccines in those aged between 6 months and 5 years is still in question and further scientific data and research will need to be undertaken so as to establish the benefits of COVID-19 immunization in the younger paediatric populous.
